# Small GTPases Rab8a and Rab11a Are Dispensable for Rhodopsin Transport in Mouse Photoreceptors

**DOI:** 10.1371/journal.pone.0161236

**Published:** 2016-08-16

**Authors:** Guoxin Ying, Cecilia D. Gerstner, Jeanne M. Frederick, Sanford L. Boye, William W. Hauswirth, Wolfgang Baehr

**Affiliations:** 1 Department of Ophthalmology and Visual Sciences, University of Utah Health Science Center, Salt Lake City, UT, 84132, United States of America; 2 Department of Ophthalmology, University of Florida, 1600 SW Archer Road, Gainesville, FL, 32610, United States of America; 3 Department of Neurobiology and Anatomy, University of Utah School of Medicine, Salt Lake City, UT, 84132, United States of America; 4 Department of Biology, University of Utah, Salt Lake City, UT, 84112, United States of America; Doheny Eye Institute, UNITED STATES

## Abstract

Rab11a and Rab8a are ubiquitous small GTPases shown as required for rhodopsin transport in *Xenopus laevis* and zebrafish photoreceptors by dominant negative (dn) disruption of function. Here, we generated retina-specific *Rab11a* (^ret^*Rab11a*) and *Rab8a* (^ret^*Rab8a*) single and double knockout mice to explore the consequences in mouse photoreceptors. Rhodopsin and other outer segment (OS) membrane proteins targeted correctly to OS and electroretinogram (ERG) responses in all three mutant mouse lines were indistinguishable from wild-type (WT). Further, AAV (adeno-associated virus)-mediated expression of dnRab11b in ^ret^*Rab11a*^-/-^ retina, or expression of dnRab8b in ^ret^*Rab8a*^-/-^ retina did not cause OS protein mislocalization. Finally, a ^ret^*Rab8a*^-/-^ retina injected at one month of age with AAVs expressing dnRab11a, dnRab11b, dnRab8b, and dnRab10 (four dn viruses on *Rab8a*^-/-^ background) and harvested three months later exhibited normal OS protein localization. In contrast to results obtained with dnRab GTPases in *Xenopus* and zebrafish, mouse Rab11a and Rab8a are dispensable for proper rhodopsin and outer segment membrane protein targeting. Absence of phenotype after expression of four dn Rab GTPases in a *Rab8a*^-/-^ retina suggests that Rab8b and Rab11b paralogs maybe dispensable as well. Our data thus demonstrate significant interspecies variation in photoreceptor membrane protein and rhodopsin trafficking.

## Introduction

Mammalian photoreceptors are polarized neurons, each consisting of an outer segment (OS), connecting cilium (CC), inner segment (IS), nucleus and synaptic terminal. The OS is a modified primary cilium that houses the phototransduction machinery, including the visual pigment rhodopsin, its G protein transducin, the target enzyme cGMP phosphodiesterase 6 (PDE6) and cGMP-gated channel subunits (CNG). Rhodopsin is the most abundant OS membrane protein [1]. Mature murine photoreceptors constantly renew OS membrane (~10% per day) by adding newly synthesized disk membranes at the OS base and removing old disks at the OS tip by phagocytosis [2;3]. As rhodopsin and other OS proteins are synthesized in the IS, photoreceptors require a sophisticated and highly directed trafficking pathway to compensate for OS protein turnover.

Much information concerning rhodopsin trafficking has been elucidated in leopard frog (*Rana berlandieri*) photoreceptors because of their large size [4;5]. Following biosynthesis and posttranslational modifications at the Golgi, rhodopsin is transported in vesicles from the trans-Golgi network (TGN) to the distal IS membrane where cargo is assembled for intraflagellar transport to the OS [6]. Rab GTPases play critical roles in guiding and docking vesicles to the target membranes. Rab proteins are small (21–25 kD), lipidated, GTP-binding and -hydrolyzing molecules present in diverse organisms from yeast to human [7]. Rab C-terminal halves contain variable regions that end with prenylation motifs signaling protein geranylgeranylation for membrane association [8]. These proteins participate in vesicular transport, docking and fusion of transport vesicles with their targets [7]. Rab8 and Rab11 each exist as two paralogs, a and b [9;10].

Rab11a and Rab8a have been shown to control rhodopsin transport in *Xenopus laevis* [11;12]. Budding of frog rhodopsin vesicles from TGN was suggested to be regulated by the GTPase Arf4 and its associated GAP, ASAP1 [4;13]. In this model, Rab11a is recruited to the vesicle surface by interacting with rhodopsin and ASAP1 during budding, and Rab11a reciprocally recruits Rab8a by recruiting Rabin 8, a Rab8 GEF (GDP-GTP exchange factor) [13;14]. Docking and fusion of rhodopsin vesicle at distal IS plasma membrane appears dependent on Rab8a, as transgenic expression of a dnRab8a (Rab8aT22N) causes massive rhodopsin-containing vesicle accumulation at the CC base and rapid retinal degeneration in *Xenopus* larva [11]. Expression of either dnRab11a or Rab11a shRNA has a similar effect in *Xenopus* retina [12]. Rab8 localization and Rab8 levels are often altered among various retina ciliopathies and are interpreted as a contributing factor of disease in mouse and zebrafish models [15–17]. In zebrafish, morpholino knockdown of Rab8a leads to rhodopsin mislocalization and OS shortening [15]. Rab8a has also been shown linked to intraflagellar transport in zebrafish through interacting with Rabaptin5 that interacts with IFT54 (intraflagellar transport protein 54 homolog) [18]. Notably, roles of Rab11 and Rab8 in rhodopsin transport are conserved across species. In *Drosophila*, Rab11 associates with rhodopsin vesicles and genetic ablation of Rab11 causes cytoplasmic accumulation of rhodopsin with failed rhabdomere morphogenesis [19], an effect that involves myosin V and an actin network [20].

In mammalian cell culture, the Rab11a-Rab8a cascade also appears to play a general role in primary ciliogenesis by delivering and docking secretory vesicles to the periciliary plasma membrane via interaction with the exocyst, as shown by disruption of Rab8a or Rab11a function by dominant negative protein expression or by RNAi knock-down [21–25]. Activated Rab8a also enters the cilium during ciliogenesis but its transport to the cilium decreases thereafter and the functional significance of Rab8-GTP in cilia is currently unknown [21;24]. In *C*. *elegans*, mutations in Rab8 suppresses formation of the membranous fans at the tip of AWB dendrite and constitutively active Rab8 results in shortened cilia, suggesting that Rab8 may inhibit cilium length but promote ciliary membrane genesis [26].

In mammals, however, the role of Rab11 and Rab8 in rhodopsin transport has not been directly tested using molecular genetics. Here we generated Rab11a KO-, Rab8a KO-, as well as Rab11a and Rab8a double KO mouse lines and found that rhodopsin and other major photo-transduction proteins traffic correctly. Further, expression of dnRab11b in *Rab11a* KO retina, or expression of dnRab8b in *Rab8a* KO retina, does not cause rhodopsin mislocalization. Simultaneously expression of dnRab8b, dnRab10, dnRab11a, and dnRab11b in retina-specific Rab8a-/- retina does not impair OS ciliary targeting of rhodopsin. These results suggest that in contrast to *Xenopus* and zebrafish, Rab11a and Rab8a, and likely their paralogs as well, are nonessential for targeting rhodopsin to the mouse outer segment. Further, as outer segments develop normally in Rab8a and11a GTPase mutants, Rab11a and Rab8a appear dispensable for photoreceptor ciliogenesis.

## Materials and Methods

### Animals

C57BL/6, flippase (FLP) and EIIa-Cre transgenic mice were obtained from The Jackson Laboratory (Bar Harbor, ME, USA). Transgenic iCre75 were generated at the University of Utah and obtained from Dr. Ching-Kang Chen [27], HRGP-Cre from Dr. Yun Z. Le (Oklahoma Health Science Center, Oklahoma City) [28], and Six3-Cre lines from Dr. Monica Vetter (University of Utah) with permission of Dr. Furuta [29]. Mice were maintained under 12-hour cyclic dark/light conditions in standard T-cages and fed Teklad Global Soy Protein-free Extruded Rodent Diet *ad libido*. Mice were sacrificed by cervical dislocation. All experimental procedures were approved by the University of Utah Institutional Animal Care and Use Committee (IACUC) and were conducted in compliance with the NIH Guide for Care and Use of Laboratory Animals.

### Generation of Retina-Specific *Rab11a*^-/-^ Mice

**Fig 1:**
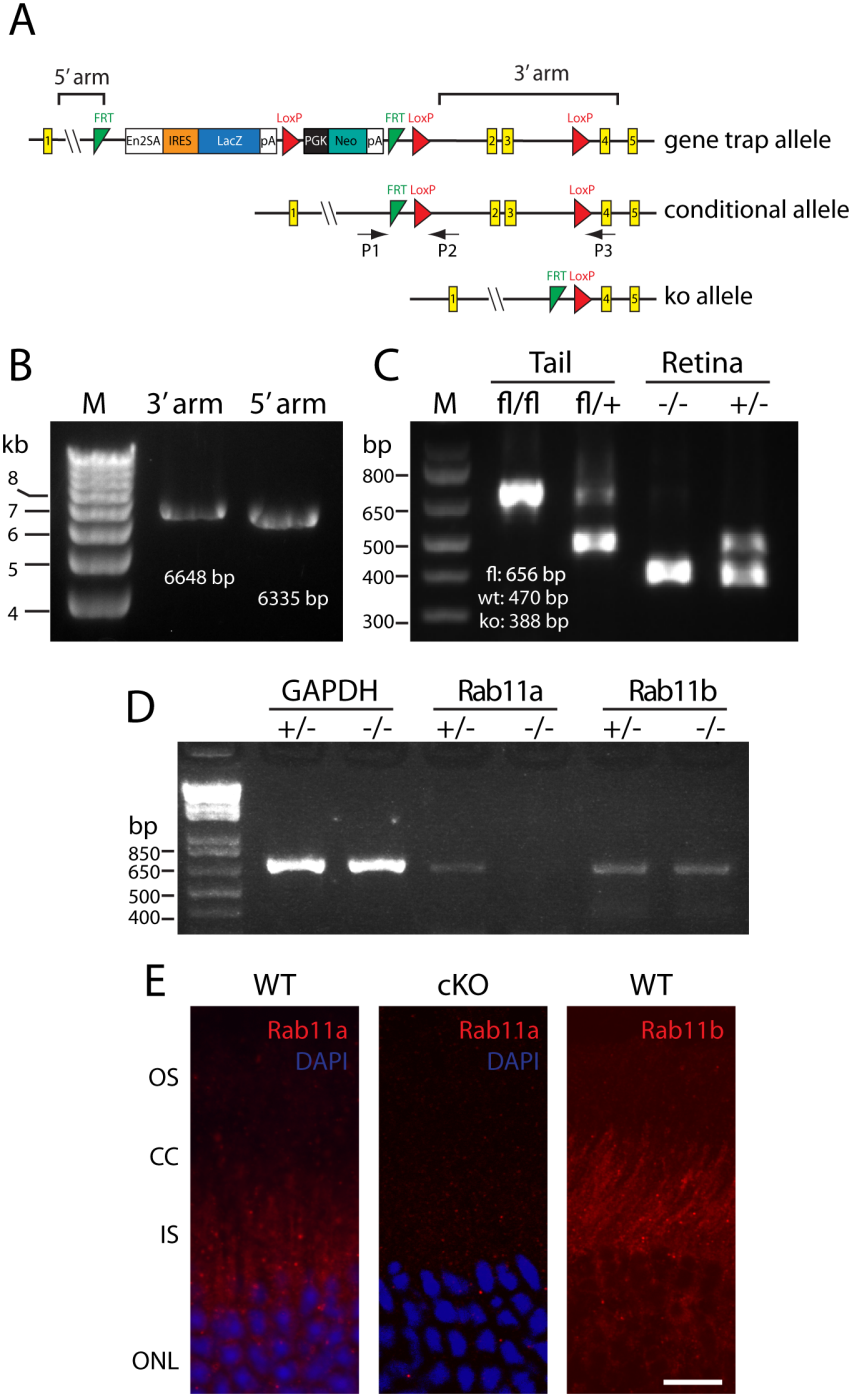
Generation of *Rab11a* retina-specific KO mouse (^ret^*Rab11a*^-/-^). (**A**) Schematic of Rab11a gene-trap (GT) allele, conditional allele and KO allele. P1, P2, P3 represent primers for genotyping wild-type (WT), conditional and KO alleles. Exons are shown as yellow rectangles. The gene trap contains a splice-acceptor (SA), an IRES site, a βGeo cassette, and a poly-A site. The gene trap is flanked by two FRT sites (green triangle), a loxP site (red triangle) at the 5’-end of the cassette and another downstream in intron 3. Location of 5’-long arm and 3’-short arm are indicated. (**B**) Long range PCR confirmation of 3’ and 5’ recombination arm in ES cells and GT animals. (**C**) PCR confirms conditional deletion of exons 2 and 3 in ^*ret*^*Rab11a*^*-/-*^ retina but not in tail. ^*ret*^*Rab11a*^*+/-*^ mouse was used as control. (**D**) *Rab11b* mRNA is not up-regulated in ^*ret*^*Rab11a*^*-/-*^ retina. +/- and -/- refer to *Rab8a* heterozygous and homozygous knockouts, respectively. GAPDH was used as internal control. (**E**) Representative retina sections showing of Rab11a and Rab11b immunostaining in retina. Rab11a is localized mainly in punctuate IS spots in WT but is negative in ^*ret*^*Rab11a*^*-/-*^ KO (center). Rab11b staining pattern is similar to Rab11a (right). Scale bar, 20 μm.

Rab11a embryonic stem (ES) cells (clone EPD0267_4_C09, C57BL/6N genetic background)) was purchased from KOMP (Knockout mouse project), the construct of which contains a gene trap (GT) cassette inserted into intron 1 and floxed exons 2 and 3 (See Fig 1A for diagram). We confirmed the gene targeting of ES cells using PCR, including 3’ and 5’ recombination arm, FRT sites and LoxP sites. ES cell blastocyst injection, generation of chimera and heterozygous (GT/+) mice was performed at the University of Utah transgenic core. Next we crossed *Rab11*^GT/+^ with flippase (FLP) mice (C57BL/6 background) to yield animals with a floxed allele (*Rab11a*^*fl/+*^). *Rab11a*^fl/fl^ were crossed with Six3-Cre animals to generate retina-specific ^ret^*Rab11a*^+/-^ and ^ret^*Rab11a*^-/-^ animals. Six3-Cre animals express Cre recombinase specifically in retina (and ventral forebrain) from embryonic day 9.5 (E9.5) [29]. The *rd8* mutation present in Eucomm C57BL/6N ES cells was removed by outcrossing to C57BL/6J mice [30].

### Generation of Retina-Specific *Rab8a*^-/-^ Mice

Sperm of *Rab8a*^fl/fl^ mice were provided by Dr. Akihiro Harada (Gunma University, Japan) [31]. *Rab8a*^fl/fl^ mice were generated by *in vitro* fertilization and blastocyst injection at the University of Utah Core. We subsequently generated *Rab8a*^-/-^ animals by crossing *Rab8a*^fl/fl^ with EIIa Cre mice [32]. Rod-, cone-, and retina-specific *Rab8a* KOs (^rod^*Rab8a*^-/-^, ^cone^*Rab8a*^-/-^, ^ret^*Rab8a*^-/-^) were generated by crossing *Rab8a*^fl/fl^ with transgenic iCre75 [27], HRGP-Cre [28], and Six3-Cre lines [29].

### Generation of Retina-Specific *Rab11a/Rab8a* Double KO Mice

Retina-specific *Rab11a* and *Rab8a* double KO mice were generated by crossing *Rab11a*^fl/fl^;*Rab8*^fl/-^ females with *Rab11a*^+/-^;*Rab8a*^+/-^;Six3-Cre^+^ males.

### Electroretinography (ERG)

ERG was performed on 5 month-old (5M) ^ret^*Rab11a*^+/-^ and ^ret^*Rab11a*^-/-^ animals, 4M ^rod^*Rab8a*^+/-^ and ^rod^*Rab8a*^-/-^, and 3M ^cone^*Rab8a*^+/-^ and ^cone^*Rab8a*^-/-^ (n = 5 each group) using an UTAS E-3000 universal electrophysiological system (LKC Technologies) as described [33]. Briefly, mice were dark-adapted overnight, anesthetized by intraperitoneal injection of ketamine (100 μg/g body weight) and xylazine (10 μg/g body weight) in 0.1M phosphate-buffered saline (PBS), and loaded onto recording platform with body temperature maintained at 37± 0.5°C. After dilating pupils with 1% tropicamide solution (Bausch & Lomb Inc., Tampa, FL), ERG responses were recorded from 5 mice of each genotype per time point. For scotopic ERG, mice were tested at intensities ranging from -40 decibels (db) (-3.4 log cds m^–2^) to 25 db (2.9 log cd s m^–2^). For photopic ERG, a rod saturating background light of 10 db (1.48 log cds m^–2^) was applied for 20 minutes before and during recording. Bactitracin ophthalmic ointment (Perrigo, Minneapolis, MN) was routinely applied to the eye to prevent infection after ERG testing, and animals were kept on heating pad until fully recovered before being returned to cages.

### Confocal Immunolocalization

Eyecups were fixed by immersion in ice-cold 4% paraformaldehyde for 2 hours in 0.1 M phosphate buffer, pH 7.4. After cryoprotection in 30% sucrose, tissues were embedded in OCT compound and frozen on dry ice. Sections (12 μm-thick) were cut using a Micron cryostat and mounted on charged Superfrost^®^ Plus slides (Fisher). Sections were washed in 0.1M PBS, blocked using 10% normal goat serum, 0.3% Triton X-100 in PBS, and incubated with primary antibodies at 4°C overnight. After washes in PBS, signals were detected with Cy3 conjugated- or Alexa 488-conjuated goat anti-rabbit/mouse secondary antibody (Jackson ImmunoResearch, Inc.) and counterstained with 1 μl/ml DAPI (4', 6-Diamidino-2-Phenylindole, Dihydrochloride, Invitrogen). Primary antibodies and dilutions were: mouse monoclonal anti-rhodopsin (1D4, Dr. Robert Molday, University of British Columbia), guanylate cyclase 1 (GC1) (IS4, Dr. Kris Palczewski, Case Western Reserve University), cyclic nucleotide gated channel alpha 1 and 3 (CNGA1/A3) (NeuroMab, UC Davis), synaptic vesicle protein 2 (SV2) (Developmental Studies Hybridoma Bank), vesicular glutamate transporter 1 (Vglut1) (NeuroMab, UC Davis), C-terminal-binding protein 2 (CTBP2 for Ribeye, BD Bioscience), synaptophysin (Sigma), Pan-plasma membrane Ca^2+^ ATPases (Pan-PMCA) (5F10, Abcam), CaM kinase II (CamKII) (6G9, Abcam); rat monoclonal anti-prominin1 (Chemicon); rabbit polyclonal anti-ML- and S-opsin (Chemicon), cone transducin gamma (Cytosignal), cone arrestin, PKCα (Abcam), Calbindin (Swant), tyrosine hydroxylase (TH) (Covance) and choline acetyltransferase (CHAT) (Chemicon). Images were captured using an Olympus Fluoview 1000 confocal microscope and 60x oil lens (1.3 NA). Some images were adjusted for brightness and contrast using Adobe Photoshop CS3.

### RT-PCR

Total RNA was extracted from retinas of two 5 month old ^ret^*Rab11a*^+/-^ and ^ret^*Rab11a*^-/-^ mice using Trizol reagents (Invitrogen), and reverse transcription (RT) was carried out using Superscript III first strand synthesis kit (Invitrogen) following manufacturer’s instructions. Full length coding sequences for Rab11a and Rab11b were PCR amplified with 28 cycles. GAPDH was used as internal control (22 cycles). PCR primers used are:Rab11a-F, Rab11a-R, Rab11b-F, Rab11b-R, GAPDH-F and GAPDH-R (Table 1).

**Table 1:** PCR primers for Rab cDNA amplification.

Rab11a	F	ATGGGCACCCGCGACGACG
	R	TT AGATGTTCTGACAGCACT GCA CC
Rab11b	F	ATGGGGACCCGGGACGACG
	R	TCACAGGCTCTGGCAGCACTGCA
Rab11a(S25N)	F	AATACTCGAGGCCACCATGGCATCAATGCAGAAGCTGATCTCAGAGGAGGACCTGGCTAGCGGCACCCGCGACGACGAGTA
	R	GCGCGGATCCAGTACTTTAGATGTTCTGACAGCACTGCACC
Rab11b(S25N)	F	AATAGCTAGCGGGACCCGGGACGACGAGT
	R	TCATAGTACTTCACAGGTTCTGGCAGCACTGCAG
Rab8b(T22N)	F	AATT CCGCGG ACCGGTCGCCACCATGGTGA
	R	GTCATCTAGACTAGT GCGGTACCGTCGACTGCAGA
Rab10(T23N)	F	AAGAGATCTGCAGCCGCAGCTGCACATGCGAAGAAGACGTACGACC
	R	TACGGTCGACTCAGCAGCATTTGCTCTTCC
GAPDH	F	GCCATCAACGACCCCTTCAT;
	R	ATGCCTGCTTCACCACCTTC

Forward (F) and reverse (R) primers used to amplify *Rab11a*, *Rab11b*, *dnRab11a*, *dnRab11b*, *dnRab8b*, *dnRab10* and *Gapdh* cDNA.

### Generation of Dominant-Negative AAV Particles

Plasmid for each canine Rab11a(S25N)-CFP was kindly provided by Yoshiyuki Wakabayashi (NICHD, NIH), human Rab11b(S25N)-pEGFPC1 by Raymond A Frizzell (University of Pittsburgh), human Rab8b(T22N)-pEGFPC1 by Dr. Johan Peränen (University of Helsinki, Finland), and human Rab10(T23N)-EGFP-pCNA3.1 by Dr. Kenneth W. Dunn (Indiana University). Rab11a(S25N) and Rab11b(S25N) with N-terminal cMyc tags were amplified by PCR and cloned into pAAV-RK-iZsgreen shuttle vector (from Dr. Tiansen Li, NEI) using restriction sites XhoI/BamHI (Rab11a) and NheI/ScaI (Rab11b). EGFP-Rab8b(T22N), EGFP-Rab10(T23N) were PCR amplified and cloned into a modified pAAV-RK shuttle vector using SacII and XbaI (Rab8b) and BglII and SalI cloning (Rab10). All clones were verified by Sanger sequencing. PCR primer sequences are listed in Table 1.

AAV was manufactured by transfection of human embryonic kidney 293 (HEK293) cell in culture using previously described methods [34]. Briefly, co-transfection of HEK293 cells with dnRab AAV vector plasmids and helper plasmid pXYZ5 (containing adenovirus helper genes, AAV2 Rep and AAV5 Cap) was followed harvest, then discontinuous iodixanol gradient ultracentrifugation and further virus purification by FPLC. AAV vector production was performed in the Retinal Gene Therapy Vector Lab at University of Florida. Genome containing virus particles were titered by quantitative real-time PCR against a known standard and resuspended in a balanced salt solution containing 0.014% Tween-20 at a concentration of ~1.0 × 10^13^ vector genomes per milliliter (vg/ml). We diluted virus to 1.0 ×10^12^ particle per milliliter and injected 1 microliter subretinally per eye for dnRab11b or dnRab8b expression. For mixed dnRab AAV injection (dnRab11a, dnRab11b, dnRab8b and dnRab10), we mixed diluted AAV in equal volumes with total titer maintained at 1.0 ×10^12^ particles/ml.

### Statistics

Data are presented as mean ± sd where *n* represents the number of mice analyzed. Statistical comparisons were done using one-way ANOVA for all experimental data. Differences were considered to be statistically significant for p< 0.05.

## Results

### Rab11a Retina Conditional Knockout Mice (^ret^*Rab11a*^-/-^)

We generated ^ret^*Rab11a*^-/-^ conditional knockouts using gene-trapped ES cell clones. In the *Rab11a* ES cell line, a gene trap (GT) cassette was inserted into intron 1 and exons 2 and 3 are flanked by loxP sites (Fig 1A). Mating with a transgenic FLP mouse line removed the GT cassette thereby generating the conditional allele. Further crossings with a Cre recombinase line under the control of a universal promoter (EIIA-Cre) [32] excised exons 2 and 3 and generated the KO allele (Fig 1A). As exons 1 and 4 are in-frame, a truncated Rab11a protein may be expressed but is predicted to be inactive and most likely degraded. We verified the gene targeting in ES cells and animals using PCR (Fig 1B). Germline deletion of Rab11a is embryonically lethal as live pups were never born (in >20 litters), as published [35]. ^ret^*Rab11a*^*-/-*^ were generated by crossing *Rab11a*^fl/fl^ with transgenic Six3-Cre mice which express Cre in retina starting at embryonic day 9.5 (E9.5). PCR analysis confirmed the deletion of exons 2 and 3 in retina but not in tail samples as expected (Fig 1C). At the RNA level, *Rab11b* mRNA expression was not upregulated In the ^ret^*Rab11a*^*-/-*^ retina (Fig 1D). Immunolocalization of Rab11a occurs predominantly in the inner segment of WT retina but is absent in the cKO retina (Fig 1E), confirming retina-specific deletion of Rab11a in ^*ret*^*Rab11a*^-/-^ mice. Antibody directed against Rab11b revealed localization in WT retina similar to Rab11a (Fig 1E).

**Fig 2:**
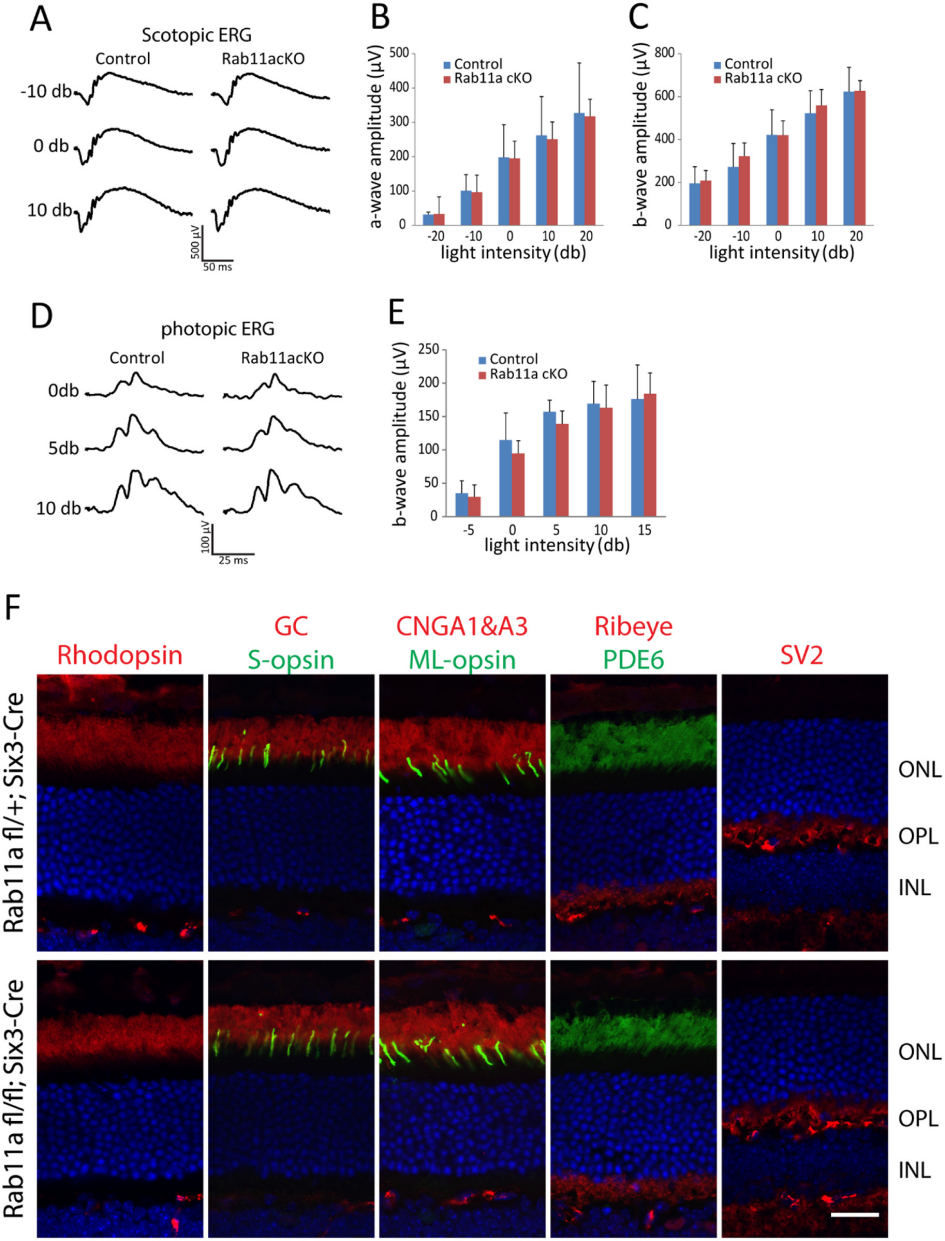
Electroretinography (ERG) and OS protein localization in adult ^*ret*^*Rab11a*^*-/-*^ mice. **(A)** Representative scotopic ERG traces at -10, 0 and 10 db from 5M-old ^ret^*Rab11a*^*+/-*^ and ^ret^*Rab11a*^*-/-*^ mice. (**B**) and (**C**) Quantification of scotopic a- and b-wave amplitude at multiple light intensities. No significant difference was observed between control and KO animals (p> 0.15, n = 5 for each group, one-way ANOVA). (**D**) Representative photopic ERG trace at 0, 5 and 10 db from 5M-old control and KO mice. (**E**) Quantification of photopic b-wave amplitude at multiple light intensities. No significant difference was observed between control and KO animals (p> 0.15, n = 5 for each group, one-way ANOVA). (**F**) Immunostaining of rhodopsin, GC1, CNGA1/A3, PDE6, S-opsin, ML-opsin, ribeye and SV2 using 3M-old animals, showing that all proteins localize correctly in KO as in heterozygous control. Scale bar, 20 μm.

To test the functional consequence of Rab11a deletion in the retina, ERGs were measured using adult ^ret^*Rab11a*^*-/-*^ mice. ^ret^*Rab11a*^*-/-*^ scotopic ERG results of 5M animals at multiple light intensities were indistinguishable from heterozygous controls (^ret^*Rab11a*^*+/-*^) (Fig 2A). Minor differences of ^ret^*Rab11a*^*-/-*^ scotopic (Fig 2B and 2C) and photopic a- or b-wave amplitudes (Fig 2D and 2E) were not statistically significant. OS proteins including rhodopsin, PDE6, GC1, CNGA1/A3, S-opsin and ML-opsin, and outer plexiform layer (OPL) proteins Ribeye and SV2, localized correctly in ^*ret*^*Rab11a*^*-/-*^ photoreceptors (Fig 2F).

### Rod and Cone Opsins Target Normally to Rab11-Deficient Outer Segments

**Fig 3:**
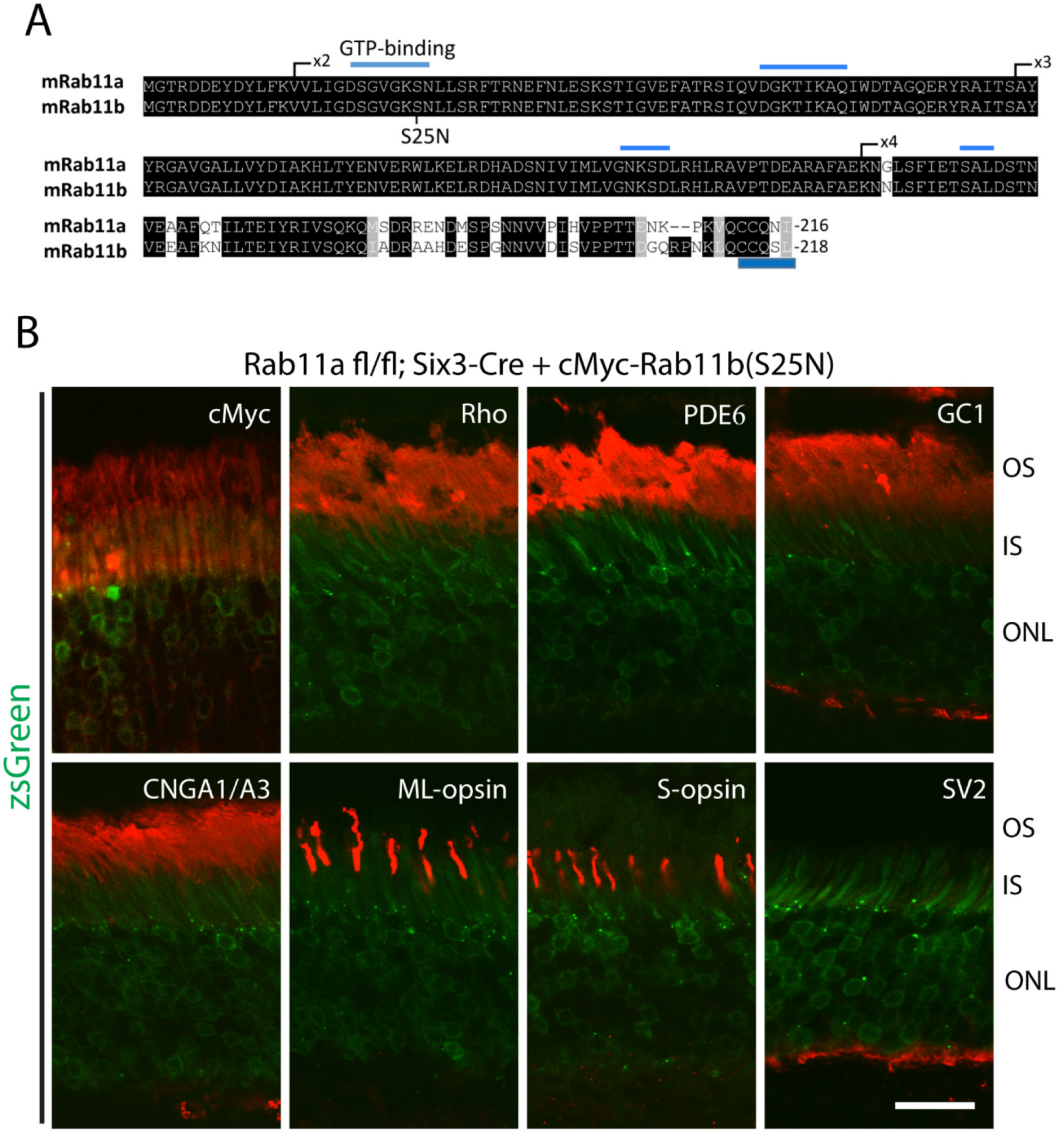
OS protein localization in adult ^ret^*Rab11a*^*-/-*^ mice injected with Rab11b(S25N) AAV. **(A**) Alignment of Rab11a and Rab11b protein sequences. One critical GTP binding domain is located in exon 2. Position of S25 in Rab11b is indicated in Rab11b. Both proteins contain geranylgeranylation motifs at the C-terminus. (**B**) Immunostaining results show that cMyc-Rab11b(S25N) localizes mainly at IS and OS (red channel, top left panel). Rhodopsin, PDE6, GC1, CNGA1/A3, ML- and S-opsin localize correctly to OS, and SV2 localizes correctly to the OPL. Green channel in all panels detect szGreen fluorescence. Scale bar, 20 μm.

The mouse genome contains two distinct Rab11 genes expressing geranylgeranylated Rab11a and Rab11b which differ only at the C-terminal variable region (overall identity 89.9%) (Fig 3A). Absence of an observed phenotype seen in ^**ret**^*Rab11a*^-/-^ retina may be attributed to Rab11a and Rab11b redundancy. Using adeno-associated virus (AAV), we expressed in ^**ret**^*Rab11a*^-/-^ retina a cMyc-tagged dnRab11b (Rab11b(S25N)), which is inactive and locked in the GDP form [36–38]. cMyc-Rab11b(S25N)-AAV was injected subretinally in 4M-old ^**ret**^*Rab11a*^-/-^ mice and heterozygous controls, and retinas were harvested two months post-injection. Presence of the reporter zsGreen, co-expressed with cMyc-Rab11b(S25N)-AAV, verified successful expression of the vector (Fig 3B). Rhodopsin, PDE6, GC1, CNGA1/A3, ML-opsin, S-opsin and OPL marker SV2 localized correctly in ^***ret***^*Rab11a*^-/-^ retina (Fig 3B) suggesting that Rab11a and 11b are dispensable for rhodopsin and OS protein targeting in mouse. We noticed that in contrast to endogenous Rab11b (Fig 1D), cMyc-Rab11b(S25N) (Fig 3B) and cMyc-Rab11a(S25N) (not shown) enter the OS, consistent with Rab11-GDP localization in *Xenopus* photoreceptor cells [12].

### Rod- and Cone-Specific Rab8a Knockout Mice

**Fig 4:**
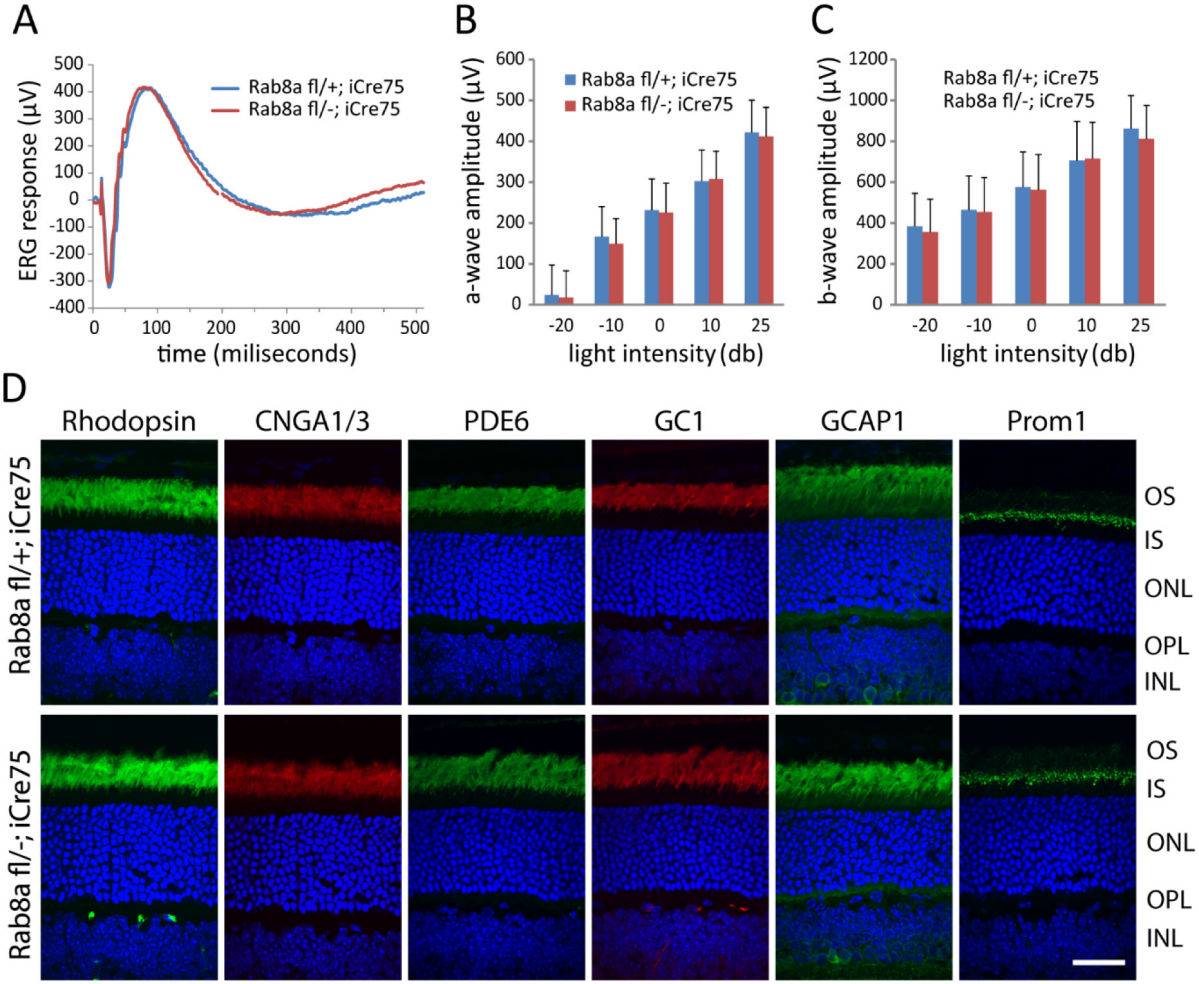
Normal ERG and rhodopsin localization in adult ^rod^*Rab8a*^-/-^ animals. **(A**) Representative scotopic ERG trace at 10 db from 4M-old ^rod^*Rab8a*^*+/-*^ and ^rod^*Rab8a*^*-/-*^ mice. (**B**) and (**C**), Quantification of scotopic a-wave and b-wave amplitudes at multiple light intensities. No significant difference was observed between control and KO animals (p> 0.17, n = 5 for each group, one-way ANOVA). (**D**), Immunostaining of rhodopsin, PDE6, GC1, CNGA1/A3, GCAP1 and prominin. All proteins localize correctly to OS. Scale bar, 20 μm.

**Fig 5:**
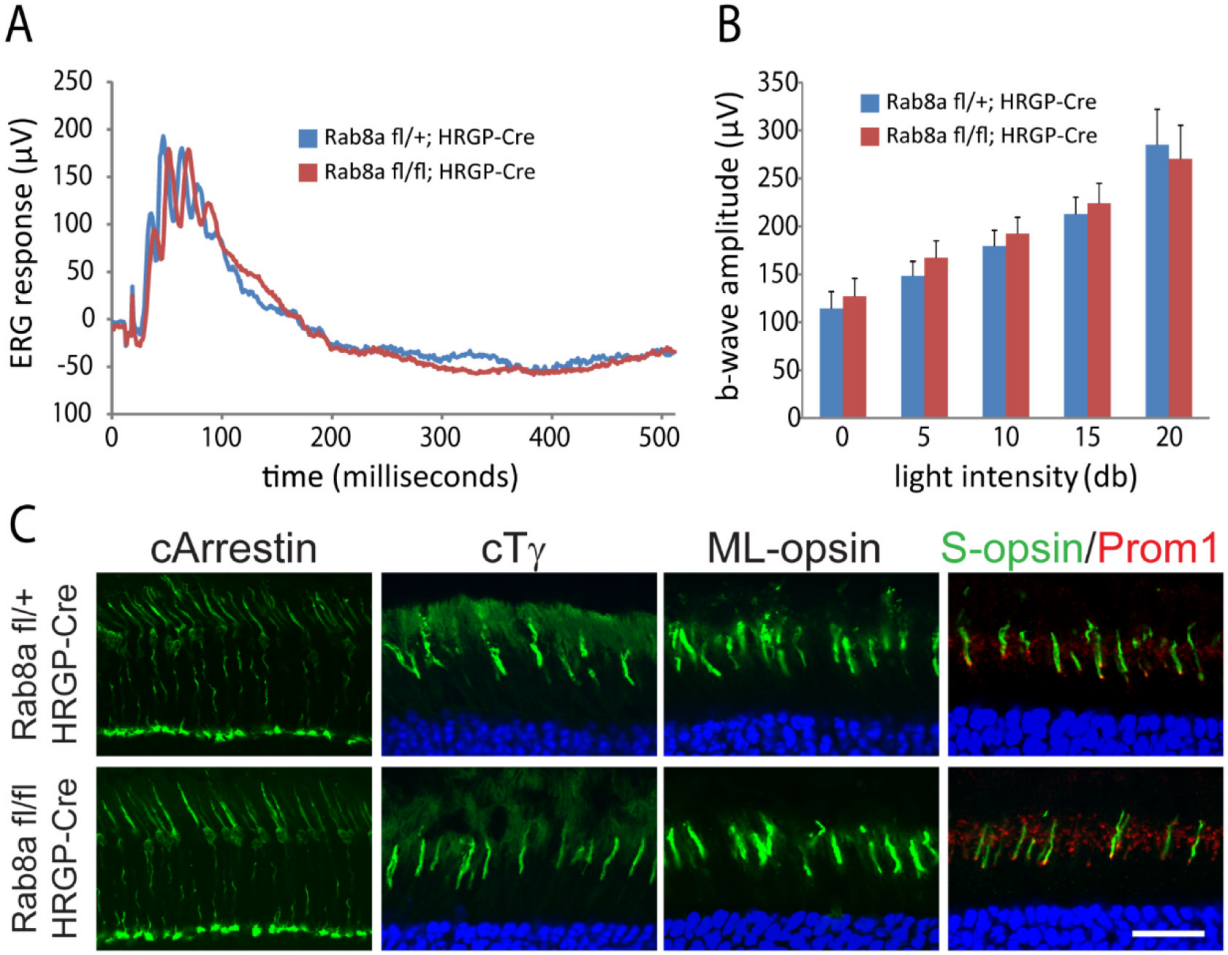
Normal ERG and opsin localization in adult ^cone^*Rab8a*^-/-^ animals. **(A**) Representative photopic ERG trace at 15 db from 3M-old ^cone^*Rab8a*^*+/-*^ and ^cone^*Rab8a*^*-/-*^ mice. (**B**) Quantification of photopic b-wave amplitude at multiple light intensities. No significant difference was observed between control and KO animals (p> 0.17, n = 5 for each group, one-way ANOVA). *C*, Immunostaining results showed that cone arrestin, cone transducin gamma, ML-opsin, S-opsin and prominin1 all localize correctly in KO as in control. Scale bar, 20 μm.

**Fig 6:**
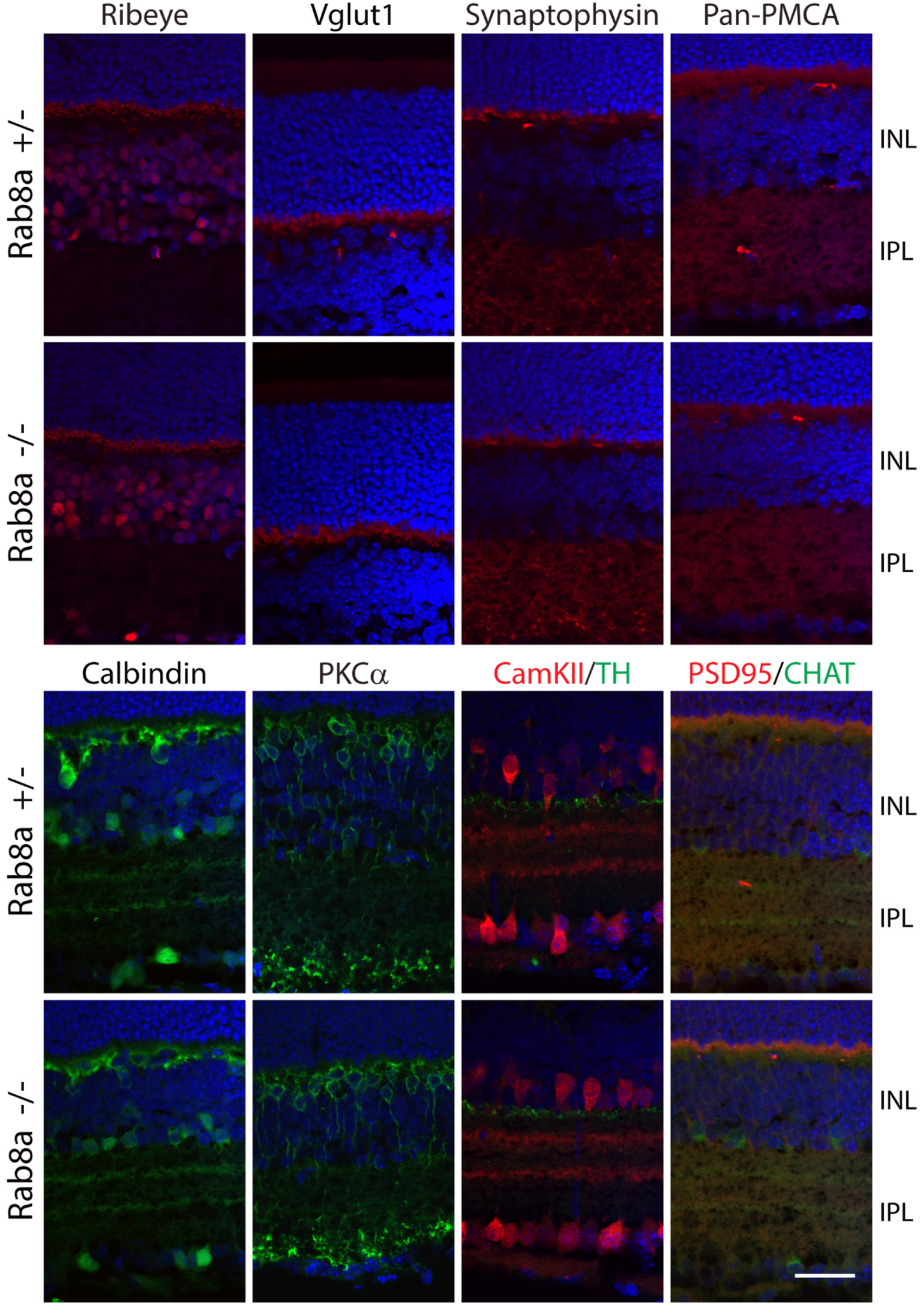
Correct localizations of OPL and inner retina proteins in Rab8a global KO mice. Immunostaining results showed that distribution of OPL proteins ribeye, Vglut1, synaptophysin, ribeye and pan-PMCA, as well as inner retina marker PKCα, calbindin, CaM kinase II (CamKII), tyrosine hydroxylase (TH) and choline acetyltransferase (CHAT), is indistinguishable between P21 *Rab8a*^*+/-*^ and *Rab8a* ^*-/-*^ controls. Scale bar, 30 μm.

A germline Rab8a knockout demonstrated that Rab8a, necessary for the apical protein localization and nutrient absorption in the small intestine, may participate in trafficking [31]. *Rab8a*^fl/fl^ mice obtained from Dr. Sato contain loxP sites in intron 1 and intron 2. Breeding with EIIA-Cre mice generated germline Rab8a knockouts (*Rab8a*^*-/-*^) in which exon 2 was deleted truncating Rab8a after exon 1 (exon 3 is out-of-frame with exon 1). Unfortunately, *Rab8a*^*-/-*^ mice died postnatally before reaching 1M due to intestinal microvillus defects [31]. ERG recordings of P16-P21 *Rab8a*^*-/-*^ mice appeared normal (results not shown). To enable tests for retina function in older mice, we generated rod-specific (^rod^*Rab8a*^*-/-*^) and cone-specific knockouts (^cone^*Rab8a*^*-/-*^) (see Methods). Scotopic ERG of ^rod^*Rab8a*^*-/-*^ and ^rod^*Rab8a*^*+/-*^ mice at various light intensities at 4M produced statistically identical responses (Fig 4A–4C). Similarly, photopic ERGs are indistinguishable from controls in 3M-old ^cone^*Rab8a*^*-/-*^ mice (Fig 5A and 5B). Rhodopsin, CNGA1/A3, PDE6, GC1, GCAP1, Prom1 in ^rod^*Rab8a*^*-/-*^ (Fig 4D) and cone Tγ, ML-opsin, and S-opsin in ^cone^*Rab8a*^*-/-*^ photoreceptors (Fig 5C) targeted normally to outer segments. Moreover, OPL proteins synaptophysin (SV2), Ribeye, pan-PMCA and Vglut1 as well as inner retina marker proteins PKCα, calbindin, tyrosine hydroxylase and choline acetyltransferase localized normally in *Rab8a*^*-/-*^ retinas (Fig 6).

### Rab8a- and Rab8b-Deficient Outer Segments Are Indistinguishable from WT

**Fig 7:**
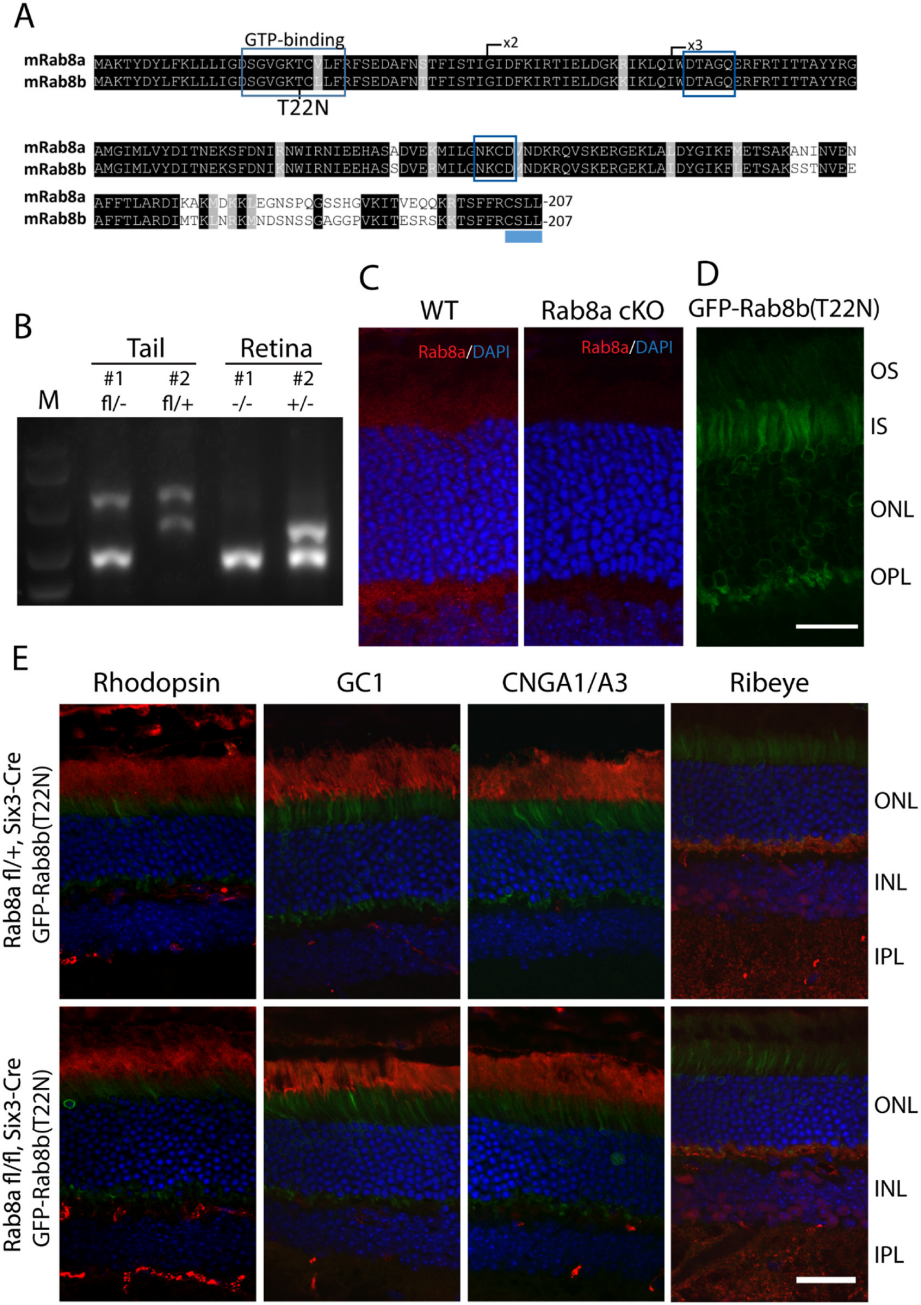
Rhodopsin localizes normally in adult Rab8a KO mice injected with Rab8b(T22N) AAV. **(A**) Alignment of Rab8a and Rab8b amino acid sequences. Exon 1 contains the GTP binding domain. Exon 2 is deleted in the germline and conditional knockouts. T22, preventing GTP binding when mutated, is shown. (**B**) Genotyping confirms deletion of exon2 in ^ret^*Rab8a*^-/-^ retinas but not tails. ^ret^*Rab8a*^+/-^ tissues were used as controls. (**C)** Immunofluorescence showed that Rab8a is mainly localized as punctate signal in IS and OPL in WT but absent in ^ret^*Rab8a*^*-/-*^ photoreceptor cells. (**D**) Rab8b(T22N) is mainly localized at IS and OPL. (**E**) Rhodopsin, GC1, CNGA1/A3 and ribeye are localized correctly in 4M-old ^*ret*^*Rab8a*^-/-^ and ^*ret*^*Rab8a*^+/-^ animals injected with dn*Rab8b* (Rab8b(T22N)) AAV. Scale bars, 20 μm (**C** and **D**) and 30 μm (**E**).

The Rab8 paralogs, Rab8a and Rab8b, are 83.6% identical (Fig 7A). As shown for Rab11, we expressed GFP-fused GDP-locked Rab8b(T22N) [39;40] virally on a retina-specific *Rab8a* knockout background. ^ret^*Rab8a*^*-/-*^ were generated by crossing *Rab8a*^*flfl-*^ with Six3-Cre mice (see Methods) [29]. Genotyping confirmed deletion of exon 2 in ^ret^*Rab8a*^*-/-*^ retina but not in tail tissues (Fig 7B). Rab8a mainly localized as punctate spots in IS and OPL in WT photoreceptor cells but was absent in ^ret^*Rab8a*^*-/-*^ photoreceptors (Fig 7C). GFP-Rab8b(T22N)-AAV was injected subretinally in 4M-old ^*ret*^*Rab8a*^*-/-*^ and control ^*ret*^*Rab8a*^*+/-*^ mice. We found that different from cMyc-Rab11b(S25N) and Rab11a(S25N) (Fig 3), GFP-Rab8b(T22N) is concentrated in the IS and is absent in the OS in adult photoreceptors (Fig 7D). Two months post-injection, ^*ret*^*Rab8a*^*-/-*^ and ^*ret*^*Rab8a*^*+/-*^ retinas were harvested to investigate OS protein localization. Rhodopsin, GC1, CNGA1/A3 and Ribeye distributed normally in Rab8b(T22N)*-*injected ^ret^*Rab8a*^-/-^ mice (Fig 7E) indicating that Rab8a and Rab8b are dispensable for transport of phototransduction and synaptic membrane proteins.

### Normal Rhodopsin Localization in Rab11a/Rab8a Double Knockout Mouse

**Fig 8:**
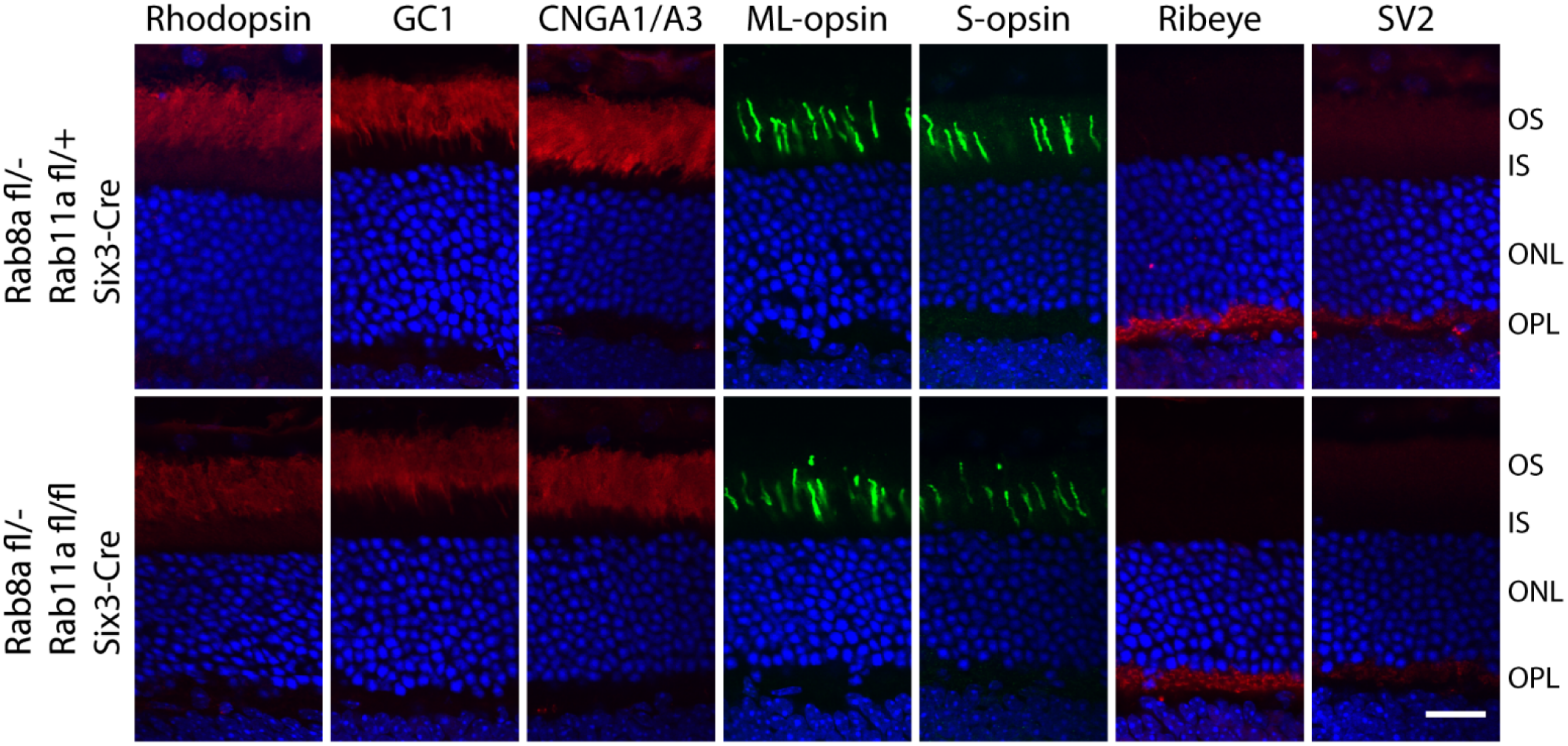
Rhodopsin localizes normally in adult Rab11a and Rab8a double KO mice. Immunostaining of rhodopsin, GC1, CNGA1/A3, PDE6, ML-opsin, S-opsin, ribeye and SV2 using 3M-old animals showed that all proteins localize correctly in dKOs (^*ret*^*Rab8a*^-/-^; ^*ret*^*Rab11a*^*-/-*^) as in controls (^*ret*^*Rab8a*^+/-^; ^*ret*^*Rab11a*^-*/-*^). Scale bar, 20 μm.

Apart from the Rab11-Rabin8-Rab8 cascade, Rab11 and Rab8 also share downstream effector proteins including the exocyst subunit Sec15 [41;42] and the molecular motor myosin Vb [43;44], suggesting there may be a possible functional redundancy between Rab11a and Rab8a polypeptides. We therefore generated ^ret^*Rab8a*^-/-ret^*Rab11a*^*-/-*^ double knockout mice and checked rhodopsin distribution. Again, rhodopsin and other transmembrane proteins destined for OS or synaptic terminals (GC1, CNGA1/A3, ML-opsin, S-opsin, Ribeye and SV2) localized correctly in the *Rab11a;Rab8a* dKOs (Fig 8).

### Expression of GFP-Rab8b(T22N), GFP-Rab10(T23N), cMyc-Rab11a(S25N) and cMyc-Rab11b(S25N) in Rab8a KO Retina Do Not Impair Rhodopsin OS Targeting

**Fig 9:**
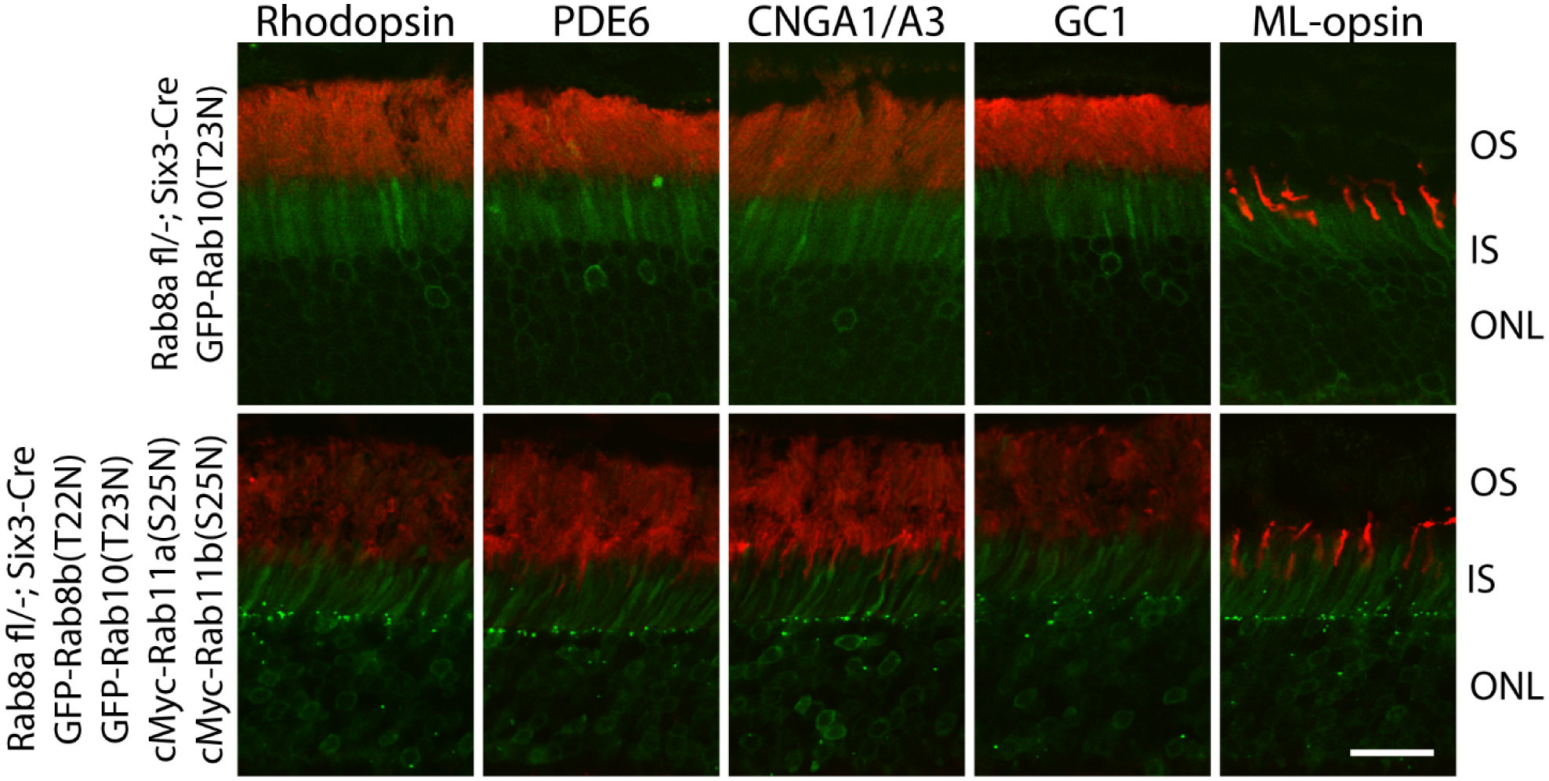
Rhodopsin correctly targets to the OS in adult Rab8a retina cKO mice injected with GFP-Rab8b(T22N), GFP-Rab10(T23N), cMyc-Rab11a(S25N) and cMYc-Rab11b(S25N) AAV. One month old ^ret^*Rab8a*^*-/-*^ mice were injected subretinally with mixed Rab11a(S25N), 11b(S25N), 8b(T22N) and 10(T23N) AAVs or Rab10(T23N) only-AAV. Three months post-injection, rhodopsin, PDE6, GC1, CNGA1/A3 and ML-opsin all localize correctly in injected animals. Scale bar, 30 μm.

As a final attempt to exclude redundancy between Rab11 and Rab8 polypeptides, we simultaneously expressed GFP- or cMyc-tagged Rab8b(T22N), Rab10(T23N), Rab11a(S25N), Rab11b(S25N) on the ^ret^*Rab8a*^-/-^ background. Rab8b(T22N) and Rab10(T23N) were GFP-tagged, and Rab11a(S25N) and Rab11b(S25N) were cMyc tagged. Rab10 was chosen because it is the closest relative of Rab8 by sequence within the Rab8 subfamily [45]. Similar to GFP-Rab8b(T22N) (Fig 7), GFP-Rab10(T23N) mainly localized at the IS and OPL (Fig 9). Three months after subretinal AAV injection into 1M-old ^*ret*^*Rab8a*^*-/-*^ mice, we found that OS proteins (rhodopsin, PDE6, GC1, CNGA1/A3, ML-Opsin) localized correctly in 4 dnRabs-injected or dnRab10 only-injected animals (Fig 9). These results suggest that members of Rab8 and Rab11 families are dispensable for rhodopsin transport to the OS.

## Discussion

Our results demonstrate that Rab8a and Rab11a are dispensable for mouse photoreceptor ciliogenesis and rhodopsin OS targeting. All tested genetic deletions and disruptions (^ret^*Rab8a*^*-/-*, ret^*Rab11a*^-/-^, ^ret^*Rab8a/11a* dKO, ^ret^*Rab8a*-/-;dn*Rab8b*, ^ret^*Rab11a*-/-;dn*Rab11b*, and ^ret^*Rab8a*^-/-^ with 4 dnRabs) had no obvious effect on rhodopsin or other phototransduction and synaptic membrane protein distribution, outer segment or synapse formation, or photoreceptor function. Our Rab8a/b knockout results are consistent with a recently generated *Rab8a/8b* dKO mouse that shows normal OS ultrastructure [46].

Previous work in frog photoreceptor cells [4;5;47–50] suggested that Rab8a and Rab11a interact directly with rhodopsin [12;14] and are components of the rhodopsin ciliary targeting complex which includes ASAP1 (ArfGAP with SH3 Domain, Ankyrin Repeat And PH Domain 1), Rab11a, Rab11 FIP3 (Rab11 family interacting protein 3), Rabin8 (GEF of Rab8) and Rab8a. During ciliary targeting, activation of Rab8a is thought to promote docking of rhodopsin-laden vesicles and their fusion to the plasma membrane at the periciliary ridge membrane. This model was supported by the observation of massive rhodopsin vesicle accumulation at CC base and faster photoreceptor degeneration in dnRab8a transgenic *Xenopus* and similar but milder rhodopsin mislocalization and photoreceptor degeneration phenotype in Rab11a shRNA or dnRab11a transgenic *Xenopus* [11;12]. Our finding that Rab11a and Rab8a, and likely their paralogs as well, are dispensable for rhodopsin OS targeting, photoreceptor ciliogenesis, and disk morphogenesis in mouse photoreceptors therefore is unexpected, but based on solid experimentation and mouse genetics.

There are several possible explanations for these interspecies discrepancies. First, the morphology of frog and mouse photoreceptors may contribute to the development of distinct rhodopsin ciliary targeting pathways. The frog rod outer segment has a much larger OS volume than mouse (~40x) and roughly 10 times more rhodopsin must be synthesized and delivered in frog rods than in mouse (700 vs. 80 molecules rhodopsin trafficking through the connecting cilium/second) [6]. It is possible that Rab11a and Rab8a are required for efficient rhodopsin transport in frog, while mouse photoreceptor cells may be able to tolerate the absence of Rab8a and Rab11a. Supporting this hypothesis, endogenous Rab8a, Rab11a or transgenic Rab8a-GFP, Rab11a-GFP are concentrated at the ciliary base as bright spots in frog photoreceptors [11;12;49], while in mouse Rab8a and Rab11a are dispersed throughout IS (Figs 1D and 7C). The absence of phenotype in Rab8a and Rab11a knockouts suggests that there may be other Rab proteins that are able to mediate rhodopsin vesicle docking and fusion (Rab redundancy). Redundancy does not originate from Rab11b and Rab8b/10, as dnRab11a in *Rab11a* KO and dnRab8b/10 in *Rab8a* KO do not cause rhodopsin mislocalization. Expression of dnRab proteins should largely, if not completely, disrupt the activity of corresponding endogenous Rab GTPase, as these constructs are effective in *in vitro* experiments [36–40;45] and they are expressed efficiently together with reporter genes in our high titer AAV injection (Figs 3B, 7D and 9). *In vitro* primary ciliogenesis assays reveal that, the phenotypes of Rab11a or Rab8a knockdowns by RNA interference only reduce the percentage of cells with intact cilia or reduce ciliary lengths [21;22;24;25]. This observation argues either that knockdowns were incomplete or that unidentified redundant Rabs partially compensate the loss of Rab11a or Rab8a in ciliary membrane trafficking. Currently, the Rab protein family comprises over 60 members (7), few of which have been investigated thoroughly using molecular genetics. To date, only mutations in Rab28 have been identified to cause human retina disease (recessive cone-rod dystrophy 18) [51;52], and defective geranylgeranylation of Rab27 was shown to be linked to choroideremia [53]. Preliminary results with a Rab28 germline knockout show that rhodopsin and OS proteins traffic normally (GY and WB, unpublished).

Alternatively, Rab8a /11a GTPases and their paralogs, may not be required at all in mouse retina for rhodopsin ciliary targeting and photoreceptor ciliogenesis. Because rhodopsin itself is a receptor for cytoplasmic dynein 1 (through binding to dynein light chain Tctex1) [54], rhodopsin vesicles have the potential of being transported from the trans-Golgi network (TGN) to the basal body by dynein motors independent of any Rab guidance since the photoreceptor microtubule track is built with the minus end anchored at the basal body, also called the microtubule organizing center [55]. Rhodopsin vesicles dissociated from the dynein complex must recruit additional proteins for plasma membrane docking and fusion to the periciliary ridge membrane. Mouse Rab8a and likely Rab8b are dispensable for docking, because *Rab8a* KO and expression of dnRab8b in *Rab8* KO, did not cause rhodopsin accumulation in the IS, an observation consistent with ultrastructural data from *Rab8a/8b* double KO mouse [46]. Further, the only detectable phenotype in Rab8a/8b germline double KO mice is the disruption of apical membrane protein transport specifically in intestinal epithelial cells. Ciliogenesis in all tested organs (including retina photoreceptors) was normal [29;46], indicating that in contrast to numerous *in vitro* data, Rab8 is not essential for general ciliary trafficking in mouse. It will be important to generate conditional Rab11a deletion in other mouse ciliated tissues (olfactory bulb, kidney) to examine whether Rab11a is generally required for ciliogenesis and ciliary trafficking in cells other than photoreceptors.

Evidence for a direct role of Rab8a or Rab11a in rhodopsin transport in frog was solely based on overexpression of dominant-negative proteins or shRNA knockdown, but not by gene knockout [11;12]. As different Rabs could share the same Rab GDP-GTP exchange factor [56], GDP-locked dominant-negative Rab11a and Rab8a may target other Rabs through sequestering the shared GEF(s). It will be necessary to verify the rhodopsin mistrafficking phenotype in Rab8a and Rab11a loss of function *Xenopus* lines by deletion with CRISPR/CAS9. Similarly, it will be important to generate Rab11a/11b double KO mice to clarify unambiguously that both Rab11a and 11b are nonessential for rhodopsin OS targeting.

Our data demonstrate that Rab11a and Rab8a are dispensable for rhodopsin transport to the mouse OS. However, a role for Rab8a and Rab11a cannot be entirely excluded, as other Rab proteins may substitute (Rab redundancy), Other membrane trafficking events may depend on Rab8a and Rab11a in mouse photoreceptors but no obvious phenotype could be observed. Rab11a has been implicated in a variety of cellular trafficking pathways with well-established roles in recycling endosome-associated membrane trafficking [9;57]. Rab8a is involved in a broad membrane trafficking pathways including endocytosis-based membrane recycling and exocytosis at the cell edge [10]. Given the protein localization pattern of Rab11 and Rab8 in IS and OPL (Figs 1 and 7), we speculate that Rab11 and Rab8 may function in endosome-related protein sorting/recycling events in the the inner segment and synaptic IS and OPL regions of mouse photoreceptors.

## References

[1] Nickell S, Park PS, Baumeister W, Palczewski K. Three-dimensional architecture of murine rod outer segments determined by cryoelectron tomography. J Cell Biol 2007 6 4; 177:917–25. 17535966 10.1083/jcb.200612010 PMC2064290

[2] Bok D, Hall MO. The role of the pigment epithelium in the etiology of inherited retinal dystrophy in the rat. J Cell Biol 1971 6; 49:664–82. 5092207 10.1083/jcb.49.3.664 PMC2108484

[3] Young RW. The renewal of photoreceptor cell outer segments. J Cell Biol 1967 4; 33:61–72. 6033942 10.1083/jcb.33.1.61 PMC2107286

[4] Wang J, Deretic D. Molecular complexes that direct rhodopsin transport to primary cilia. Prog Retin Eye Res 2014 1; 38:1–19. 10.1016/j.preteyeres.2013.08.004 24135424 PMC3883129

[5] Wang J, Deretic D. The Arf and Rab11 effector FIP3 acts synergistically with ASAP1 to direct Rabin8 in ciliary receptor targeting. J Cell Sci 2015 4 1; 128:1375–85. 10.1242/jcs.162925 25673879 PMC4379727

[6] Pearring JN, Salinas RY, Baker SA, Arshavsky VY. Protein sorting, targeting and trafficking in photoreceptor cells. Prog Retin Eye Res 2013 9; 36:24–51. 10.1016/j.preteyeres.2013.03.002 23562855 PMC3759535

[7] Stenmark H. Rab GTPases as coordinators of vesicle traffic. Nat Rev Mol Cell Biol 2009 8; 10:513–25. 10.1038/nrm2728 19603039

[8] Farnsworth CC, Seabra MC, Ericsson LH, Gelb MH, Glomset JA. Rab geranylgeranyl transferase catalyzes the geranylgeranylation of adjacent cysteines in the small GTPases Rab1A, Rab3A, and Rab5A. Proc Natl Acad Sci U S A 1994 12 6; 91:11963–7. 7991565 10.1073/pnas.91.25.11963 PMC45356

[9] Welz T, Wellbourne-Wood J, Kerkhoff E. Orchestration of cell surface proteins by Rab11. Trends Cell Biol 2014 7; 24:407–15. 10.1016/j.tcb.2014.02.004 24675420

[10] Peranen J. Rab8 GTPase as a regulator of cell shape. Cytoskeleton (Hoboken) 2011 10; 68:527–39. 21850707 10.1002/cm.20529

[11] Moritz OL, Tam BM, Hurd LL, Peranen J, Deretic D, Papermaster DS. Mutant rab8 Impairs docking and fusion of rhodopsin-bearing post-Golgi membranes and causes cell death of transgenic Xenopus rods. Mol Biol Cell 2001 8; 12:2341–51. 11514620 10.1091/mbc.12.8.2341 PMC58598

[12] Reish NJ, Boitet ER, Bales KL, Gross AK. Nucleotide bound to rab11a controls localization in rod cells but not interaction with rhodopsin. J Neurosci 2014 11 5; 34:14854–63. 10.1523/JNEUROSCI.1943-14.2014 25378153 PMC4220021

[13] Mazelova J, Astuto-Gribble L, Inoue H, Tam BM, Schonteich E, Prekeris R, et al Ciliary targeting motif VxPx directs assembly of a trafficking module through Arf4. EMBO J 2009 2 4; 28:183–92. 10.1038/emboj.2008.267 19153612 PMC2637330

[14] Wang J, Morita Y, Mazelova J, Deretic D. The Arf GAP ASAP1 provides a platform to regulate Arf4- and Rab11-Rab8-mediated ciliary receptor targeting. EMBO J 2012 10 17; 31:4057–71. 10.1038/emboj.2012.253 22983554 PMC3474927

[15] Bachmann-Gagescu R, Phelps IG, Stearns G, Link BA, Brockerhoff SE, Moens CB et al The ciliopathy gene cc2d2a controls zebrafish photoreceptor outer segment development through a role in Rab8-dependent vesicle trafficking. Hum Mol Genet 2011 10 15; 20:4041–55. 10.1093/hmg/ddr332 21816947 PMC3177654

[16] Westfall JE, Hoyt C, Liu Q, Hsiao YC, Pierce EA, Page-McCaw PS et al Retinal degeneration and failure of photoreceptor outer segment formation in mice with targeted deletion of the Joubert syndrome gene, Ahi1. J Neurosci 2010 6 30; 30:8759–68. 10.1523/JNEUROSCI.5229-09.2010 20592197 PMC2923804

[17] Murga-Zamalloa CA, Desai NJ, Hildebrandt F, Khanna H. Interaction of ciliary disease protein retinitis pigmentosa GTPase regulator with nephronophthisis-associated proteins in mammalian retinas. Mol Vis 2010; 16:1373–81. 20664800 PMC2905641

[18] Omori Y, Zhao C, Saras A, Mukhopadhyay S, Kim W, Furukawa T et al Elipsa is an early determinant of ciliogenesis that links the IFT particle to membrane-associated small GTPase Rab8. Nat Cell Biol 2008 4; 10:437–44. 10.1038/ncb1706 18364699

[19] Satoh AK, O'Tousa JE, Ozaki K, Ready DF. Rab11 mediates post-Golgi trafficking of rhodopsin to the photosensitive apical membrane of Drosophila photoreceptors. Development 2005 4; 132:1487–97. 15728675 10.1242/dev.01704

[20] Li BX, Satoh AK, Ready DF. Myosin V, Rab11, and dRip11 direct apical secretion and cellular morphogenesis in developing Drosophila photoreceptors. J Cell Biol 2007 5 21; 177:659–69. 17517962 10.1083/jcb.200610157 PMC2064211

[21] Nachury MV, Loktev AV, Zhang Q, Westlake CJ, Peranen J, Merdes A et al A core complex of BBS proteins cooperates with the GTPase Rab8 to promote ciliary membrane biogenesis. Cell 2007 6 15; 129:1201–13. 17574030 10.1016/j.cell.2007.03.053

[22] Knodler A, Feng S, Zhang J, Zhang X, Das A, Peranen J et al Coordination of Rab8 and Rab11 in primary ciliogenesis. Proc Natl Acad Sci U S A 2010 4 6; 107:6346–51. 10.1073/pnas.1002401107 20308558 PMC2851980

[23] Yoshimura S, Egerer J, Fuchs E, Haas AK, Barr FA. Functional dissection of Rab GTPases involved in primary cilium formation. J Cell Biol 2007 7 30; 178:363–9. 17646400 10.1083/jcb.200703047 PMC2064854

[24] Westlake CJ, Baye LM, Nachury MV, Wright KJ, Ervin KE, Phu L et al Primary cilia membrane assembly is initiated by Rab11 and transport protein particle II (TRAPPII) complex-dependent trafficking of Rabin8 to the centrosome. Proc Natl Acad Sci U S A 2011 2 15; 108:2759–64. 10.1073/pnas.1018823108 21273506 PMC3041065

[25] Feng S, Knodler A, Ren J, Zhang J, Zhang X, Hong Y et al A Rab8 guanine nucleotide exchange factor-effector interaction network regulates primary ciliogenesis. J Biol Chem 2012 5 4; 287:15602–9. 10.1074/jbc.M111.333245 22433857 PMC3346093

[26] Mukhopadhyay S, Lu Y, Shaham S, Sengupta P. Sensory signaling-dependent remodeling of olfactory cilia architecture in C. elegans. Dev Cell 2008 5; 14:762–74. 10.1016/j.devcel.2008.03.002 18477458 PMC2442577

[27] Li S, Chen D, Sauve Y, Mc Candles J, Chen YJ, Chen C-K. Rhodopsin-iCre transgenic mouse line for Cre-mediated rod-specific gene targeting. Genesis 2005; 41:73–80. 15682388 10.1002/gene.20097

[28] Le YZ, Ash JD, Al-Ubaidi MR, Chen Y, Ma JX, Anderson RE. Targeted expression of Cre recombinase to cone photoreceptors in transgenic mice. Mol Vis 2004 12 27; 10:1011–8. 15635292

[29] Furuta Y, Lagutin O, Hogan BL, Oliver GC. Retina- and ventral forebrain-specific Cre recombinase activity in transgenic mice. Genesis 2000 2; 26:130–2. 10686607

[30] Mattapallil MJ, Wawrousek EF, Chan CC, Zhao H, Roychoudhury J, Ferguson TA et al The Rd8 mutation of the Crb1 gene is present in vendor lines of C57BL/6N mice and embryonic stem cells, and confounds ocular induced mutant phenotypes. Invest Ophthalmol Vis Sci 2012; 53:2921–7. 10.1167/iovs.12-9662 22447858 PMC3376073

[31] Sato T, Mushiake S, Kato Y, Sato K, Sato M, Takeda N et al The Rab8 GTPase regulates apical protein localization in intestinal cells. Nature 2007 7 19; 448:366–9. 17597763 10.1038/nature05929

[32] Lakso M, Pichel JG, Gorman JR, Sauer B, Okamoto Y, Lee E et al Efficient in vivo manipulation of mouse genomic sequences at the zygote stage. Proc Natl Acad Sci U S A 1996 6 11; 93:5860–5. 8650183 10.1073/pnas.93.12.5860 PMC39152

[33] Jiang L, Zhang H, Dizhoor AM, Boye SE, Hauswirth WW, Frederick JM et al Long-term RNA interference gene therapy in a dominant retinitis pigmentosa mouse model. Proc Natl Acad Sci U S A 2011 11 8; 108:18476–81. 10.1073/pnas.1112758108 22042849 PMC3215008

[34] Zolotukhin S, Potter M, Zolotukhin I, Sakai Y, Loiler S, Fraites TJ et al Production and purification of serotype 1, 2, and 5 recombinant adeno-associated viral vectors. Methods 2002 10; 28:158–67. 12413414 10.1016/s1046-2023(02)00220-7

[35] Yu S, Yehia G, Wang J, Stypulkowski E, Sakamori R, Jiang P et al Global ablation of the mouse Rab11a gene impairs early embryogenesis and matrix metalloproteinase secretion. J Biol Chem 2014 11 14; 289:32030–43. 10.1074/jbc.M113.538223 25271168 PMC4231680

[36] Butterworth MB, Edinger RS, Silvis MR, Gallo LI, Liang X, Apodaca G et al Rab11b regulates the trafficking and recycling of the epithelial sodium channel (ENaC). Am J Physiol Renal Physiol 2012 3 1; 302:F581–F590. 22129970 10.1152/ajprenal.00304.2011 PMC3353647

[37] Best JM, Foell JD, Buss CR, Delisle BP, Balijepalli RC, January CT et al Small GTPase Rab11b regulates degradation of surface membrane L-type Cav1.2 channels. Am J Physiol Cell Physiol 2011 5; 300:C1023–C1033. 10.1152/ajpcell.00288.2010 21248079 PMC3093944

[38] Silvis MR, Bertrand CA, Ameen N, Golin-Bisello F, Butterworth MB, Frizzell RA et al Rab11b regulates the apical recycling of the cystic fibrosis transmembrane conductance regulator in polarized intestinal epithelial cells. Mol Biol Cell 2009 4; 20:2337–50. 10.1091/mbc.E08-01-0084 19244346 PMC2669039

[39] Hattula K, Furuhjelm J, Arffman A, Peranen J. A Rab8-specific GDP/GTP exchange factor is involved in actin remodeling and polarized membrane transport. Mol Biol Cell 2002 9; 13:3268–80. 12221131 10.1091/mbc.E02-03-0143 PMC124888

[40] Chen S, Liang MC, Chia JN, Ngsee JK, Ting AE. Rab8b and its interacting partner TRIP8b are involved in regulated secretion in AtT20 cells. J Biol Chem 2001 4 20; 276:13209–16. 11278749 10.1074/jbc.M010798200

[41] Guo W, Roth D, Walch-Solimena C, Novick P. The exocyst is an effector for Sec4p, targeting secretory vesicles to sites of exocytosis. EMBO J 1999 2 15; 18:1071–80. 10022848 10.1093/emboj/18.4.1071 PMC1171198

[42] Wu S, Mehta SQ, Pichaud F, Bellen HJ, Quiocho FA. Sec15 interacts with Rab11 via a novel domain and affects Rab11 localization in vivo. Nat Struct Mol Biol 2005 10; 12:879–85. 16155582 10.1038/nsmb987

[43] Lapierre LA, Kumar R, Hales CM, Navarre J, Bhartur SG, Burnette JO et al Myosin vb is associated with plasma membrane recycling systems. Mol Biol Cell 2001 6; 12:1843–57. 11408590 10.1091/mbc.12.6.1843 PMC37346

[44] Roland JT, Kenworthy AK, Peranen J, Caplan S, Goldenring JR. Myosin Vb interacts with Rab8a on a tubular network containing EHD1 and EHD3. Mol Biol Cell 2007 8; 18:2828–37. 17507647 10.1091/mbc.E07-02-0169 PMC1949367

[45] Babbey CM, Ahktar N, Wang E, Chen CC, Grant BD, Dunn KW. Rab10 regulates membrane transport through early endosomes of polarized Madin-Darby canine kidney cells. Mol Biol Cell 2006 7; 17:3156–75. 16641372 10.1091/mbc.E05-08-0799 PMC1483048

[46] Sato T, Iwano T, Kunii M, Matsuda S, Mizuguchi R, Jung Y et al Rab8a and Rab8b are essential for several apical transport pathways but insufficient for ciliogenesis. J Cell Sci 2014 1 15; 127:422–31. 10.1242/jcs.136903 24213529 PMC3898603

[47] Wang J, Morita Y, Mazelova J, Deretic D. The Arf GAP ASAP1 provides a platform to regulate Arf4- and Rab11-Rab8-mediated ciliary receptor targeting. EMBO J 2012 10 17; 31:4057–71. 10.1038/emboj.2012.253 22983554 PMC3474927

[48] Mazelova J, Ransom N, Astuto-Gribble L, Wilson MC, Deretic D. Syntaxin 3 and SNAP-25 pairing, regulated by omega-3 docosahexaenoic acid, controls the delivery of rhodopsin for the biogenesis of cilia-derived sensory organelles, the rod outer segments. J Cell Sci 2009 6 15; 122:2003–13. 10.1242/jcs.039982 19454479 PMC2723154

[49] Deretic D, Huber LA, Ransom N, Mancini M, Simons K, Papermaster DS. rab8 in retinal photoreceptors may participate in rhodopsin transport and in rod outer segment disk morphogenesis. J Cell Sci 1995; 108:215–24. 7738098 10.1242/jcs.108.1.215

[50] Deretic D, Puleo-Scheppke B, Trippe C. Cytoplasmic domain of rhodopsin is essential for post-Golgi vesicle formation in a retinal cell-free system. J Biol Chem 1996; 271:2279–86. 8567690 10.1074/jbc.271.4.2279

[51] Roosing S, Rohrschneider K, Beryozkin A, Sharon D, Weisschuh N, Staller J et al Mutations in RAB28, Encoding a Farnesylated Small GTPase, Are Associated with Autosomal-Recessive Cone-Rod Dystrophy. Am J Hum Genet 2013 7 11; 93:110–7. 10.1016/j.ajhg.2013.05.005 23746546 PMC3710761

[52] Riveiro-Alvarez R, Xie YA, Lopez-Martinez MA, Gambin T, Perez-Carro R, Avila-Fernandez A et al New mutations in the RAB28 gene in 2 Spanish families with cone-rod dystrophy. JAMA Ophthalmol 2015 2; 133:133–9. 10.1001/jamaophthalmol.2014.4266 25356532 PMC4351871

[53] Seabra MC, Ho YK, Anant JS. Deficient geranylgeranylation of Ram/Rab27 in choroideremia. J Biol Chem 1995 10 13; 270:24420–7. 7592656 10.1074/jbc.270.41.24420

[54] Tai AW, Chuang JZ, Bode C, Wolfrum U, Sung CH. Rhodopsin's carboxy-terminal cytoplasmic tail acts as a membrane receptor for cytoplasmic dynein by binding to the dynein light chain Tctex-1. Cell 1999 6 25; 97:877–87. 10399916 10.1016/s0092-8674(00)80800-4

[55] Troutt LL, Burnside B. Microtubule polarity and distribution in teleost photoreceptors. J Neurosci 1988 7; 8:2371–80. 3249231 10.1523/JNEUROSCI.08-07-02371.1988 PMC6569541

[56] Yoshimura S, Gerondopoulos A, Linford A, Rigden DJ, Barr FA. Family-wide characterization of the DENN domain Rab GDP-GTP exchange factors. J Cell Biol 2010 10 18; 191:367–81. 10.1083/jcb.201008051 20937701 PMC2958468

[57] Kelly EE, Horgan CP, McCaffrey MW. Rab11 proteins in health and disease. Biochem Soc Trans 2012 12 1; 40:1360–7. 10.1042/BST20120157 23176481

